# Spontaneous acute subdural hematoma in dengue fever: Case report and review of the literature

**DOI:** 10.1016/j.ijscr.2022.107512

**Published:** 2022-08-13

**Authors:** Andre Marolop Pangihutan Siahaan, Steven Tandean, Edwin Batara Saragih, Bahagia Willibrordus Maria Nainggolan

**Affiliations:** a Department of Neurosurgery, Faculty of Medicine, Universitas Sumatera Utara, Medan, Indonesia; b Division of Neurosurgery, Rumah Sakit Vita Insani, Siantar, Indonesia; c Department of Neurosurgery, Medical Faculty, Universitas Sumatera Utara, Medan, Indonesia

**Keywords:** Acute subdural hematoma, Case report, Dengue fever

## Abstract

**Introduction and importance:**

A very uncommon dengue fever consequence is subdural hematoma. IgG positivity, increased AST, and ALT levels may all be risk factors for bleeding in dengue fever patients.

**Case presentation:**

We report the case of a 65-year-old man who presented with dengue fever symptoms and developed altered consciousness and focal neurological deficits. The findings of the tests showed thrombocytopenia, increased AST and ALT, positive anti-dengue IgG, and subdural hematoma on brain imaging. The urgent operations were completed satisfactorily.

**Clinical discussion:**

Dengue-related intracerebral haemorrhage is still a complicated condition. Thrombocytopenia and leukopenia are the first symptoms that point to dengue. Some risk factors, such as thrombocytopenia and increased AST and ALT, have been identified as bleeding factors in dengue fever. For a possible intracerebral haemorrhage, radiological imaging should be performed. In an emergency neurosurgery setting, thrombocyte administration could be used to monitor thrombocytopenia.

**Conclusion:**

Subdural hematoma is a possible dengue fever complication. If the patient's symptoms with thrombocytopenia and elevated liver enzymes indicate the possibility of intracranial haemorrhage, immediate radiological imaging should be performed.

## Highlights

• Elevated AST and ALT as a risk factor of intracranial bleeding in dengue fever
• Immediate radiological imaging to the patients with positive IgG and intracranial haemorrhage symptoms.
• Emergency surgical intervention should be conducted depending on patient's status with thorough care in hematologic profile.

## 1 Introduction

Dengue fever is a tropical infection caused by the dengue virus [1]. Although dengue has several complications, subdural hematoma is a rare complication in dengue fever [2]. Thrombocytopenia and higher aspartate transaminase (AST) and alanine transaminase (ALT) in dengue have been shown to be part of the risk factors for plasma leakage [3], [4]. There are currently no precise guidelines for the timing of radiological imaging and neurosurgical surgery for subdural hematoma in dengue [5].

We report the case of a 65-year-old man who presented with dengue fever symptoms and, after two days in the hospital, developed altered consciousness and focal neurological deficits. The test results revealed thrombocytopenia, elevated AST and ALT, and positive anti-dengue IgG, and brain imaging revealed a subdural hematoma. Emergency surgical procedures were successfully performed done after monitoring the thrombocytopenia. This work is reported according to the SCARE criteria and the revised 2020 SCARE guidelines [6].

## 2 Case presentation

**Fig. 1:**
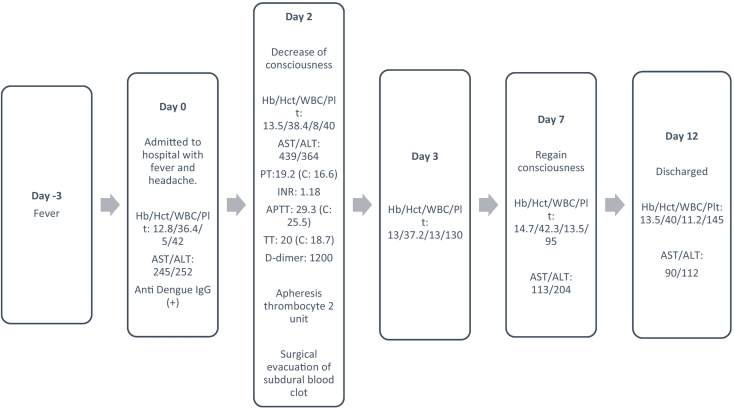
Timeline of the reported case.

A 65-year-old man went to the emergency room with three days of extensive body pain and high fever (maximum recorded temperature of 39 degrees Celsius). He was previously a healthy man without a history of cardiovascular or neurological conditions, and he had never undergone surgery. On physical examination, he was discovered to be conscious, with a blood pressure of 135/70 mmHg and a heart rate of 108 beats per minute. No active bleeding or rash was observed. The results of a general physical examination were normal. Initial laboratory results were as follows: haemoglobin (Hb) 12.8 g/L, haematocrit (Hct) 36.4 %, white blood cells (WBC) 5 K/L, platelet count (PC) 42 K/L, aspartate aminotransferase (AST) 245 U/L, and alanine aminotransferase (ALT) 252 U/L. Dengue was verified by the presence of dengue-NS1-antigen and anti-dengue antibody (IgG). Fluid treatment per WHO recommendations and symptomatic medications were administered. The timeline of the reported incident is depicted in Fig. 1.

**Fig. 2:**
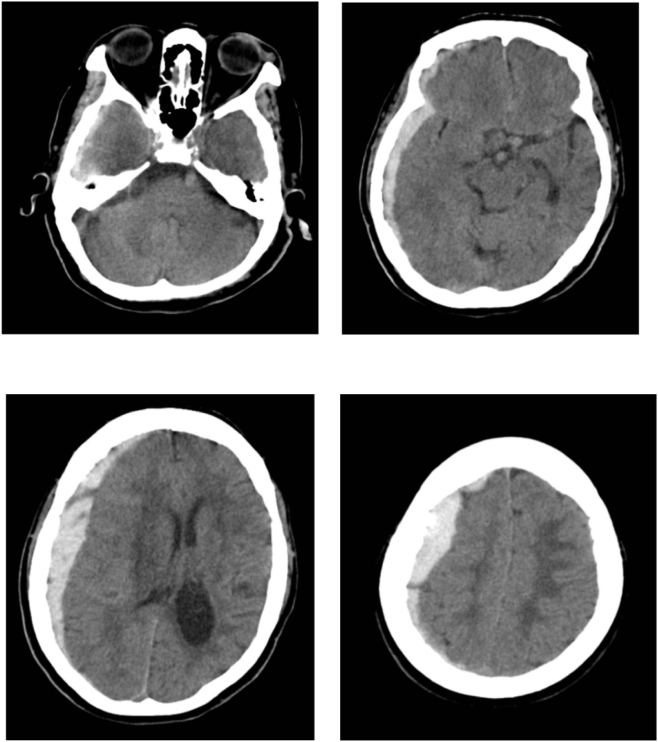
CT scan of the head without contrast. On the right hemisphere, a hyperdense, crescentic-shaped lesion was observed, consistent with subdural haemorrhage.

Two days after admission, the patient experienced an abrupt loss of consciousness with a Glasgow Coma Scale (GCS) score of 9/15 (E2V5M2), accompanied by left hemiparesis and right eye anisocoria. A head CT scan revealed a 1 cm thick subdural hematoma on the left side, with a 5 mm midline displacement (Fig. 2).

**Fig. 3:**
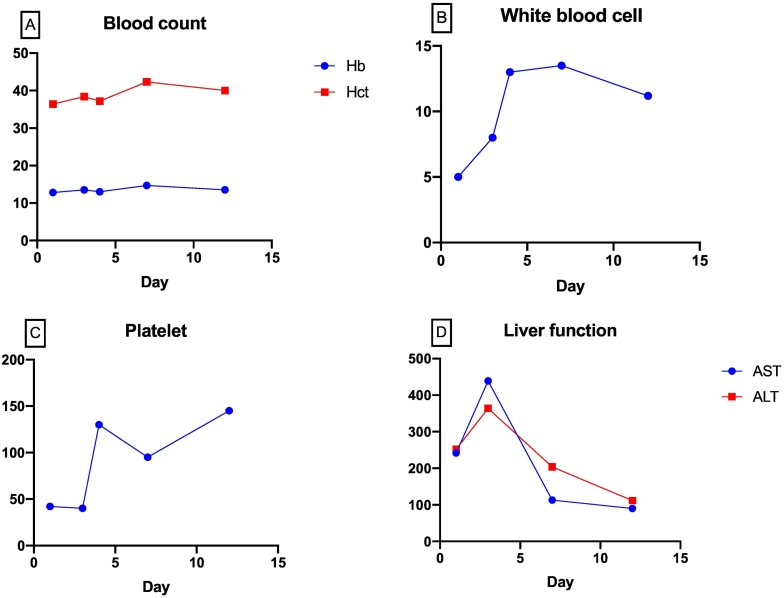
Trend in laboratory results. There were no significant variations in haemoglobin and haematocrit (A). There was a considerable increase in white blood cell count following surgical operation, which decreased few days later (B). Prior to the operation, there was no substantial increase in platelet count (C). The AST and ALT levels were elevated for the first two days after admission, but had not returned to normal by the time of discharge (D).

The chest X-ray and abdomen ultrasound were normal. Compared to initial laboratory results (42 K/L), laboratory examinations revealed a little drop in platelet count (40 K/L, Fig. 3A), but a notable rise in AST and ALT levels (439 and 364, respectively, Fig. 3B). Routine haemorrhagic screening tests (PT, APTT, and TT) were normal, but the D-dimer result was elevated (1200).

A general neurosurgeon performed a craniotomy as a result of increasing loss of consciousness and evidence of intracranial bleeding. Two units of apheresis thrombocyte were administered, one prior to surgery and the remainder during surgery. There was no evidence of cortical bleeding, indicating that the source of the bleeding was a cerebral bridging vein. Following surgery, the post-operative period was found to be uneventful. The patient improved gradually over the next few days and regained full consciousness (GCS 15/15) five days following surgery. On day three, the platelet count was >100,000/mm3, and until day twelve, the AST/ALT level gradually decreased. Twelve days after his admission, he was sent home in fully alert state and no motoric weakness, but he complained mild headache. On follow up, one month after his discharge, he was neurologically normal with no deficits.

## 3 Discussion

Dengue virus is a flavivirus that is transmitted by urban-dwelling *Aedes aegyptus* and *Aedes albopictus*. There are four recognized serotypes of dengue virus, DENV 1–4 [5], [7]. Neurological signs are uncommon, occurring in <1 % of patients with dengue fever, but intracranial haemorrhage may develop due to thrombocytopenia [8], [9]. Intracerebral haemorrhage associated with dengue remains complex, however the postulated cause is mostly associated with haemostatic problems. Vascular activity, capillary leakage, increased fibrinolysis, and release of mediators with bleeding could be resulted from the infection of dengue virus [4], [10].

The initial suspicion of dengue is usually based on the demonstration of thrombocytopenia and leukopenia [5], [11]. NS1 antigen test is commonly used to detect the virus in the early phase while IgG is detectable at high levels during secondary infection and IgM is higher at early primary infection and lower during secondary infection [12]. In this case, the test results showed positive NS1 antigen and positive IgG but negative IgM, showed a secondary infection of dengue. Unfortunately, due to limitation of facility in our hospital, we did not perform PCR as confirmatory test.

Since ICH in patients with dengue is rarely reported, it is very helpful to prevent ICH in patients with dengue before both the surgery and medical interventions can no longer be preserved [5]. A study in India found biphasic fever patterns, haemoconcentration, thrombocytopenia (<50.000/mm3), and increased ALT had a positive predicting value and a negative predicting value, 70 % and 75 % respectively, in predicting spontaneous bleeding in dengue [4]. Talukdar et al. reported three factors that are associated with plasma leakage, including Body Mass Index(BMI) ≥25.0 kg/m^2^, platelet count <100.000/mm^3^ and elevated AST and ALT (≥100 U/I) [3]. A study reported a cerebral haemorrhage with thrombocytopenia (40.000/mm^3^) and elevated AST and ALT (666 and 312, respectively) [13]. However, a study reported spontaneous subdural hematoma secondary to dengue fever without thrombocytopenia [1]. Another study found that three patients with positive IgG emergency surgery and all of them died while two patients with negative IgG did not have ICH requiring surgery and survived [5]. Therefore, secondary infections may be associated with severe ICH. In this case, the patient had thrombocytopenia, increased AST and ALT, and positive IgG antibody test. Elevated AST and ALT can be the risk factors of intracranial haemorrhage in dengue fever while positive IgG may be associated with severity of ICH. To demonstrate these aspects, though, more study needs to be done.

So far, there are no guidelines on when to perform a brain computed tomography (CT) scan. Patients with suspected ICH with dengue fever may have symptoms such as headache, fever, and vomiting [5]. Since it is not possible to advise screening Computed Tomography (CT) head in every dengue patients, CT should be considered only there is high index of suspicion based on clinical findings [14]. A study reported patients with secondary dengue infections with early detection of IgG and negative IgM are at higher risk of ICH carrying poor prognosis and should be monitored with lower thresholds for diagnostic CT of the brain when suspicion of ICH present [5]. Thus, based on study we found, we did CT immediately after the patient were showing a suspicion symptoms of ICH and had positive IgG antibody test.

Neurosurgical intervention in ICH patients with dengue remains questionable due to vascular disorders, coagulation disorders, and platelet dysfunction which require correction of platelets and other coagulation parameters by blood transfusion [5]. Many factors affect the outcome of patients [15], [16]. Timely surgical interventions performed within 8 h of bleedings were also associated with improved outcomes [17]. The advised recommendation for platelet counts for neurosurgical methods is 100 × 10^9^/L. [18] In dengue patients, it is hard to preserve the perioperative platelet levels as recommended.Furthermore, it is proven that a perioperative platelet count below 100 × 10^9^/L in patients who failed to respond to platelet transfusions had a higher risk of haemorrhagic complication after surgery [19]. A recent study reported a successful emergency subdural hematoma evacuation in dengue patients with thrombocytopenia, while platelet counts were monitored and transfused [20]. However, Sam et al. reported that two surgical patients died due to excessive bleeding during the procedure [5]. We did the emergency evacuation in this patient due to the subdural hematoma while monitoring the platelet counts by administering 2 units of apheresis thrombocytes. Post-operative platelet counts were gradually increased to normal and the patient regained consciousness 5 days after surgery and was sent home 5 days later.

## 4 Conclusion

Subdural hematoma is a rare complication of dengue fever. Elevated AST and ALT can be predictor for bleeding in dengue fever. Radiological imaging should be done if the test results show positive IgG and intracranial haemorrhage symptoms. Neurosurgical intervention should be done immediately while maintaining the platelet counts.

## Funding

None.

## Ethical approval

None declared.

## Consent for publication

Written informed consent was obtained from the patient for publication of this case report and accompanying images. A copy of the written consent is available for review by the Editor-in-Chief of this journal on request.

## Guarantor

Andre Marolop Pangihutan Siahaan, who had access to the data and is in charge of the publication decision, accepts full responsibility for the work and/or study's conduct.

## Provenance and peer review

Not commissioned, externally peer reviewed.

## CRediT authorship contribution statement

Andre Marolop Pangihutan Siahaan, Steven Tandean, Edwin Batara Saragih = Patient management (including surgery), study concept, data collection.

Bahagia Willibrordus Maria Nainggolan and Andre Marolop Pangihutan Siahaan = Writing- original draft preparation.

Bahagia Willibrordus Maria Nainggolan and Andre Marolop Pangihutan Siahaan = Editing and writing.

Andre Marolop Pangihutan Siahaan, Steven Tandean, Edwin Batara Saragih = senior author and manuscript reviewer.

All the authors read and approved the final manuscript.

## Declaration of competing interest

None.
